# A Two-Stage Ensemble Machine Learning Pipeline for Breast Cancer Diagnosis from Digital Mammograms

**DOI:** 10.3390/jimaging12080378

**Published:** 2026-08-12

**Authors:** Fernando Martín-Rodríguez, Carmen Freire-Bouza, Mónica Fernández-Barciela, Ainhoa Morales-Fernández, María Marante-Boado

**Affiliations:** atlantTTic Research Center for Telecommunication Technologies, University of Vigo, 36310 Vigo, Spain; fmartin@uvigo.es (F.M.-R.); carmen.freire@alumnado.uvigo.gal (C.F.-B.); monica.barciela@uvigo.es (M.F.-B.); ainhoa.morales@uvigo.es (A.M.-F.)

**Keywords:** breast cancer diagnosis, digital mammography, computer-aided diagnosis (CAD), machine learning, deep learning, convolutional neural networks (CNNs), ensemble learning

## Abstract

Breast cancer is the most common cancer among women, and early detection through mammography is essential for reducing mortality. Artificial intelligence can support radiologists by improving diagnostic accuracy. To develop and evaluate a two-stage ensemble machine learning pipeline for breast cancer diagnosis from digital mammograms. The proposed framework combines image preprocessing, multiple convolutional neural networks trained under different conditions, and a second-stage classifier that integrates the CNN outputs. Several machine learning models and feature selection techniques were evaluated using publicly available mammography datasets. Results: The ensemble approach consistently outperformed the individual CNN models. The MLP classifier achieved the best overall balance between precision and recall, while the heuristic fusion method provided the highest sensitivity. Feature selection reduced model complexity while maintaining comparable performance, and cross-validation confirmed the robustness of the proposed methodology. Combining complementary information from multiple CNNs with classical machine learning improves diagnostic performance and provides a robust framework for computer-aided breast cancer diagnosis. The proposed two-stage ensemble offers an effective and interpretable approach for mammographic breast cancer classification. A demonstration application incorporating Grad-CAM explainability further supports its potential use as a clinical decision-support tool.

## 1. Introduction

Breast cancer is the most common type of cancer worldwide according to the World Health Organization (WHO), with more than 2.3 million cases reported in 2022 [1]. Nearly 1 in 12 women will develop breast cancer during their lifetime. In addition, it affects women across all age groups after puberty, with the probability increasing significantly after the age of 40. Approximately half of all breast cancer cases occur in women with no identifiable risk factors other than gender and age.

Since 1980, mortality rates in developed countries have been reduced by 40% due to the implementation of regular mammographic screening programs in the most affected age groups. Therefore, early detection and treatment are essential to reducing the number of deaths. Furthermore, there are significant disparities in patient survival rates between high-income countries and low- and middle-income countries, largely due to the high cost of diagnosis, which requires the involvement of radiologists with specialized expertise in this field.

Currently, thanks to recent advances in artificial intelligence, and more specifically, in machine learning, interest in the development of automatic and semi-automatic diagnostic support systems has increased considerably. These tools are not intended to replace healthcare professionals, but rather to complement their work, optimize their time, and improve productivity, contributing to faster and better-informed clinical decision-making.

This work presents a system for the analysis of digital mammograms using machine learning techniques. A complete platform trained on publicly available datasets is developed, including a preprocessing stage and two modeling phases. In the first phase, several convolutional neural networks (CNNs) [2] trained with different datasets extract relevant information from the images. In the second phase, their outputs are combined as feature vectors to perform the final classification. After evaluating several methods (MLP, SVM, BT, XGBoost, and an heuristic method), the novel heuristic one achieved an F_1_ score and recall higher than 0.75. The area under the curve (AUC) was also computed, showing values higher than 0.80. MLP [3] offered also interesting results. A demonstration application was also implemented.

The application of convolutional neural networks to computer-aided diagnosis of breast cancer from mammographic images has been an active research area in recent years. In a systematic review by Nasser and Yusof [4], deep learning-based CAD systems for breast cancer are analyzed. Better results are for systems that use genomic and/or histopathological information. For the particular case of mammograms, we found some examples. In ref. [5], CNNs are combined with generative adversarial networks (GANs), achieving 0.88 AUC; ref. [6] it focuses on segmentation given a manually introduced ROI, achieving 0.87 IoU; and ref. [7] it applies CNN on extracted ROIs from mammograms, training CNNs where an adversarial network is used to augment training data, with a maximum reported accuracy of 0.85. The authors of ref. [8] obtain impressive results with 0.98 recall using multiple modalities for input images (combining mammography with ultrasound, MRI, and tomosynthesis).

Other interesting work is that from Dehghan Rouzi et al. [9], proposing a CAD ensemble system integrating multiple pretrained deep networks (EfficientNet, Xception, MobileNetV2, InceptionV3, and ResNet50) via a consensus-adaptive weighting method, achieving an accuracy of 0.95 on image crops (selected ROIs). Nevertheless, F scores on whole mammograms were not so impressive. Similarly, Shah et al. [10] combined EfficientNet, AlexNet, ResNet, and DenseNet in an optimized ensemble, demonstrating that the complementary feature extraction capabilities of heterogeneous architectures lead to superior diagnostic performance compared to any single model. In a broader survey following PRISMA guidelines, Masud et al. [11] analyzed 50 studies published between 2018 and 2025 and confirmed that hybrid and ensemble models consistently outperform standalone CNN or classical ML approaches in mammogram classification. A common thread in these works is the reliance on large pretrained architectures and transfer learning. By contrast, the present work proposes two lightweight CNNs: the first one is a novel architecture trained from scratch, and the second one is the well-known ResNet-18 architecture, trained via transfer learning. Six differently trained CNNs are used in a first stage and then combined through an ensemble strategy based on a classical classifier. This approach achieves competitive sensitivity while significantly reducing computational requirements.

Other interesting works are: ref. [12], where the authors focus in classifying known lesions, ref. [13], where different models are trained on different datasets obtaining, heterogeneous results ranging from 0.79 to 0.90 AUC, and ref. [14], where the importance of categorizing images attending to breast density is studied as a method to enhance the identification.

The main contribution of this paper is the development of a complete two-stage ensemble machine learning pipeline for breast cancer diagnosis from digital mammograms. Unlike approaches based solely on a single deep learning model, the proposed system combines the predictive capabilities of N independently trained convolutional neural networks with a second-stage classifier that integrates their outputs as a feature vector for final decision-making. This strategy improves diagnostic performance, achieving recall higher than 0.80, which is particularly relevant in medical diagnosis where minimizing false negatives is critical. In addition, the work includes a dedicated preprocessing methodology for heterogeneous DICOM mammography images and the implementation of a demonstration application capable of providing a quantitative breast cancer risk score directly from mammographic images. The application also incorporates explainability through the Grad-CAM technique, highlighting the image regions that most strongly support the final diagnostic decision. This improves interpretability and increases the practical applicability of the proposed system for clinical decision support.

The remainder of this paper is organized as follows. Section 2 presents the datasets and the proposed methodology. Section 3 reports the experimental results and their analysis. Finally, Section 4 discusses the main findings, compares the evaluated methods, highlights the limitations of the proposed approach, and concludes with future research directions.

## 2. Materials and Methods

### 2.1. Datasets

The starting point of this study was a dataset published on the Kaggle platform [15]. This dataset consists of a large collection of classified mammograms in DICOM format [16] compiled by the Radiological Society of North America (RSNA). These data come from different hospitals and have been acquired by a mix of different equipment. Depending on the type of study and the associated diagnosis, the distribution of the images is shown below (Table 1).

Four types of images were found: CC: “cranial–caudal” (top view), ML: “mediolateral” (from the center), MLO: “mediolateral–oblique” (oblique view from the center), and LM: “lateromedial” (from the outside toward the center).

In Table 1, it can be observed that the most common image types are CC and MLO. In addition, the dataset is clearly highly imbalanced. The direct application of any machine learning model would result in a system that tends to prioritize the negative (healthy) class.

To alleviate the imbalance problem, another public dataset was obtained from [17]. Again it is a heterogeneous dataset coming from different origins. This second dataset contains only positive cases: 1324 positive cases (631 CC and 693 MLO).

From this initial collection, the following working datasets were created:

- BLD-CC: Balanced CC dataset for training: 1017 samples per class; 180 samples per class reserved for testing.
- BLD-MLO: Balanced MLO dataset for training: 1091 samples per class; 192 samples per class reserved for testing.
- BLD-MIX: Combination of the two previous datasets for training: 2108 samples per class; 372 samples per class reserved for testing.
- AUG-CC: Imbalanced CC dataset (to be balanced using data oversampling): 2320 healthy and 1017 sick cases for training; 180 samples per class reserved for testing.
- AUG-MLO: Imbalanced MLO dataset: 2308 healthy and 1091 sick cases for training; 192 samples per class reserved for testing.
- AUG-MIX: Combination of the two previous augmented datasets for training: 4628 healthy and 2108 sick cases; 372 samples per class reserved for testing.

These six datasets are summarized in Table 2 below. Slight differences in balanced datasets are due to maintaining each patient wholly in training or testing.

Although the dataset is fully anonymized, each patient is assigned a unique identifier that is used as the prefix for all corresponding data files. This naming convention organizes the dataset by patient and enables a simple and reliable partitioning strategy, ensuring that data from the same patient are never included in both the training and test sets.

Data oversampling consists of generating synthetic samples to balance an imbalanced dataset, especially when the ratio between the majority and minority classes is moderate (less than 3:1). In many cases, this is achieved using interpolation between nearby samples (SMOTE algorithm). In this case, since the data consist of images, oversampling is performed by simple random repetition of minority-class examples until the number of samples matches that of the majority class. Oversampling is done only in the training part of the dataset leaving the test part untouched to avoid overestimating results.

### 2.2. Pipeline Scheme

**Figure 1:**
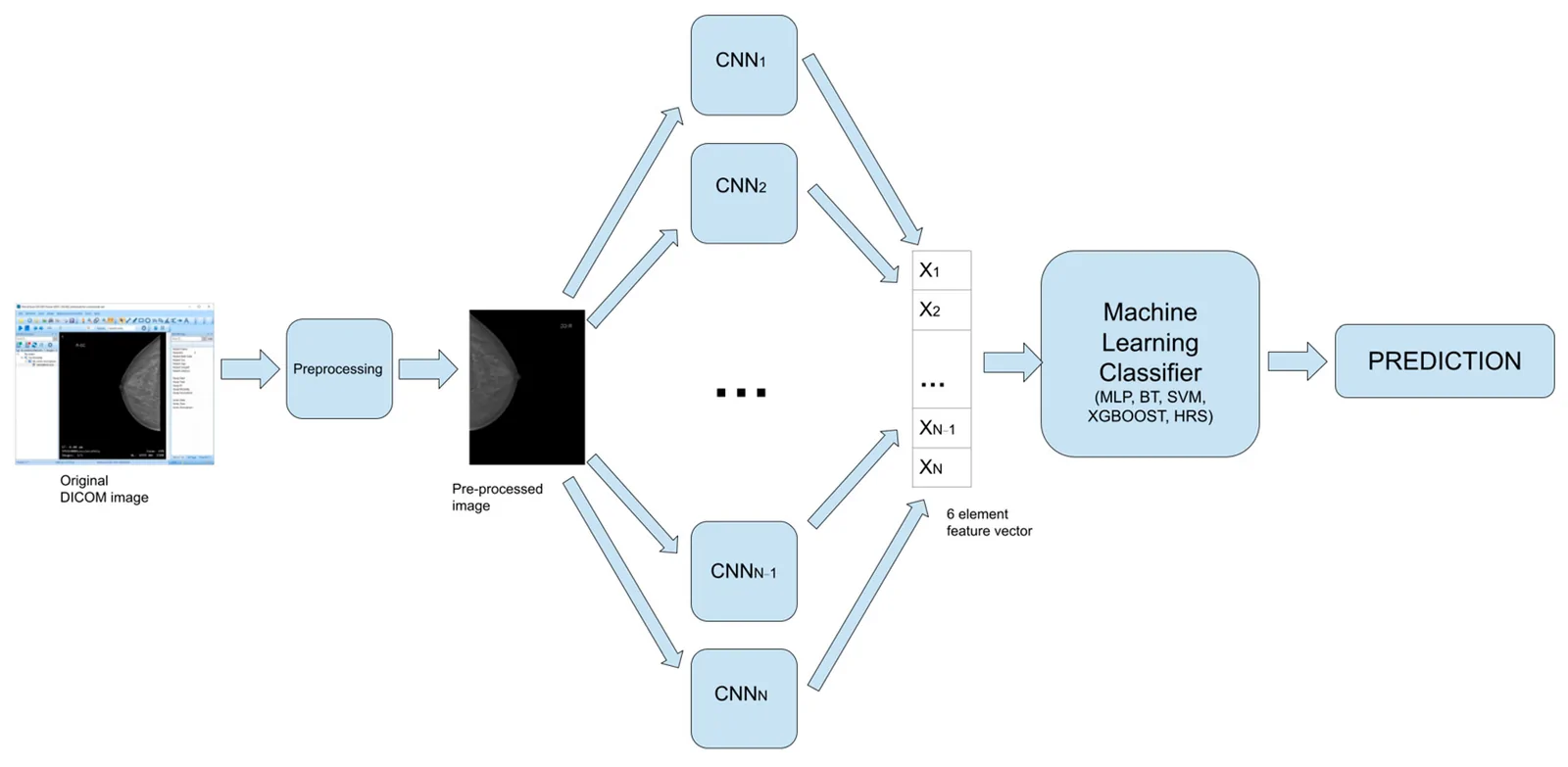
Pipeline general diagram.

This subsection presents the overall architecture of the proposed system. The final pipeline was established based on the performance obtained at each stage during the development process. The resulting block diagram is shown in Figure 1.

As illustrated in Figure 1, the input image first undergoes the preprocessing stage, after which it is analyzed by **N** independently trained CNNs. Each network is trained to perform the diagnostic classification task described in Section 2.4. The resulting outputs are then combined into a feature vector that serves as the input to a second-stage machine learning classifier, trained independently of the CNNs, to produce the final diagnostic decision. Several combinations of first-stage CNNs and classical machine learning algorithms were evaluated and compared to identify the most effective strategy for this final classification stage.

### 2.3. Preprocessing Stage

Preprocessing is necessary because the dataset contains a mixture of images from different sources. For example, the number of bits per pixel in DICOM files may be 10, 12, or 16. Image size can also vary, and CNN-based networks require fixed-size input images.

The applied preprocessing consists of the following operations:

- Conversion to floating-point numerical format through an affine transformation that ensures maximum contrast (minimum value equal to 0.0 or black, maximum value equal to 1.0 or white).
- When necessary, horizontal mirroring along the X-axis of the image to ensure that the significant information is always located on the left side.
- Nonlinear contrast enhancement using a pointwise nonlinear power-law filter: *y = x^n^*, with *n* = 1.2 if the total sum of pixel values is less than 50% of the maximum possible value, and n = 2 otherwise.
- Resizing to a fixed size of 384 × 512 pixels. Most available files have an aspect ratio of 3:4 = 0.75 (*ar* = width/height). In some cases, this value is smaller, and the image is resized without distortion to a height of 512 pixels. Then, blank columns are added to the right side. If ar were greater than 0.75, the opposite procedure would be applied: resizing to a width of 384 pixels and adding blank rows at the top.

**Figure 2:**
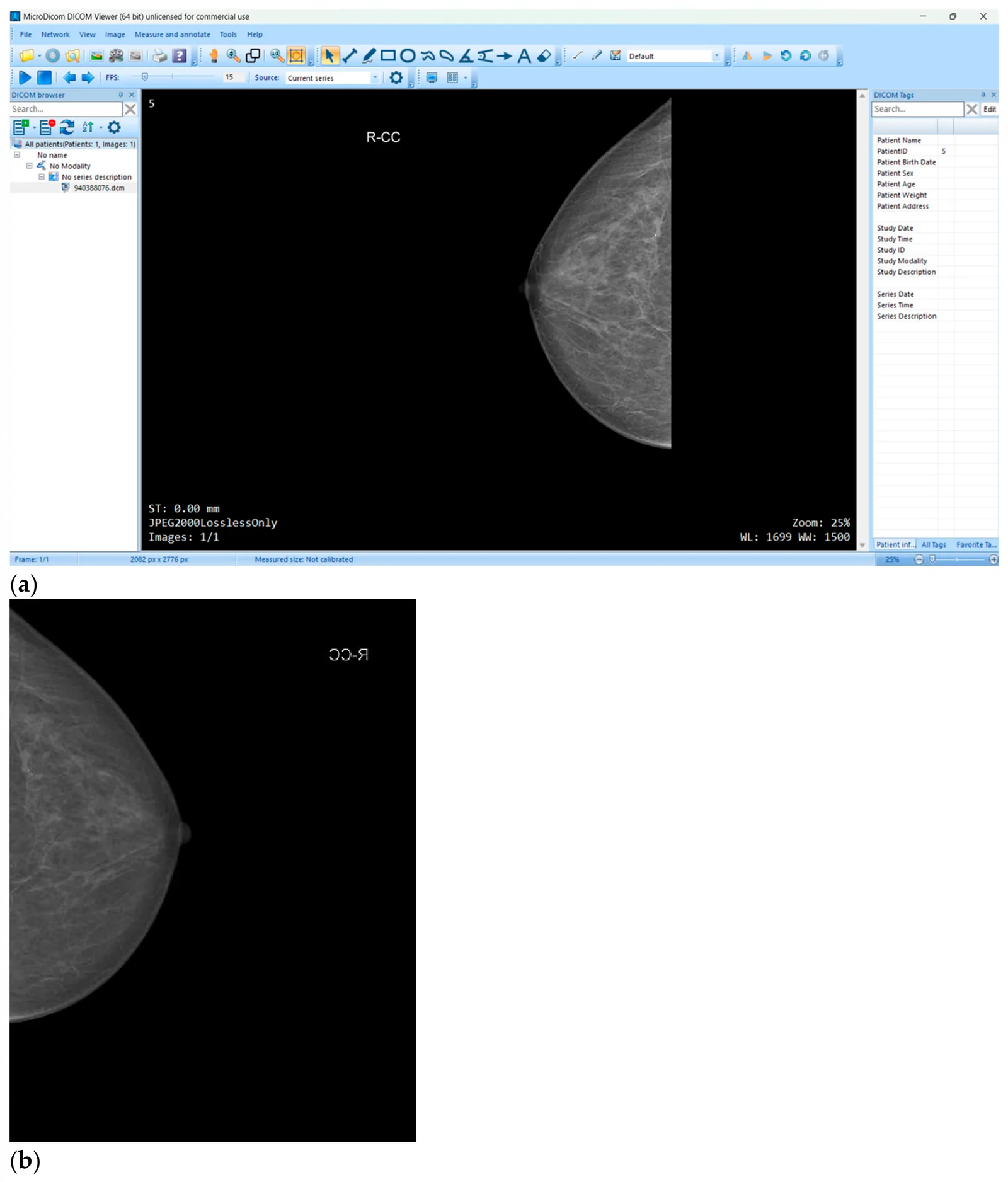
Digital mammogram: (**a**) original DICOM file displayed by a DICOM viewer (microdicom), (**b**) preprocessed image.

Figure 2 shows a real mammogram displayed using a DICOM viewer (original image) and the same image after the complete preprocessing procedure.

### 2.4. Convolutional Neural Networks

A CNN is a cascade of filter banks and nonlinear operations followed by a multilayer perceptron (sometimes consisting of only a single layer). The perceptron is a set of individual neurons connected to all numerical values from the previous layer (fully connected layers).

The main purpose of the initial stages is to extract relevant information from the input images in order to facilitate the classification task in the final stages. The training algorithm includes the filter parameters (impulse responses), allowing the feature extraction process to be optimized.

**Figure 3:**
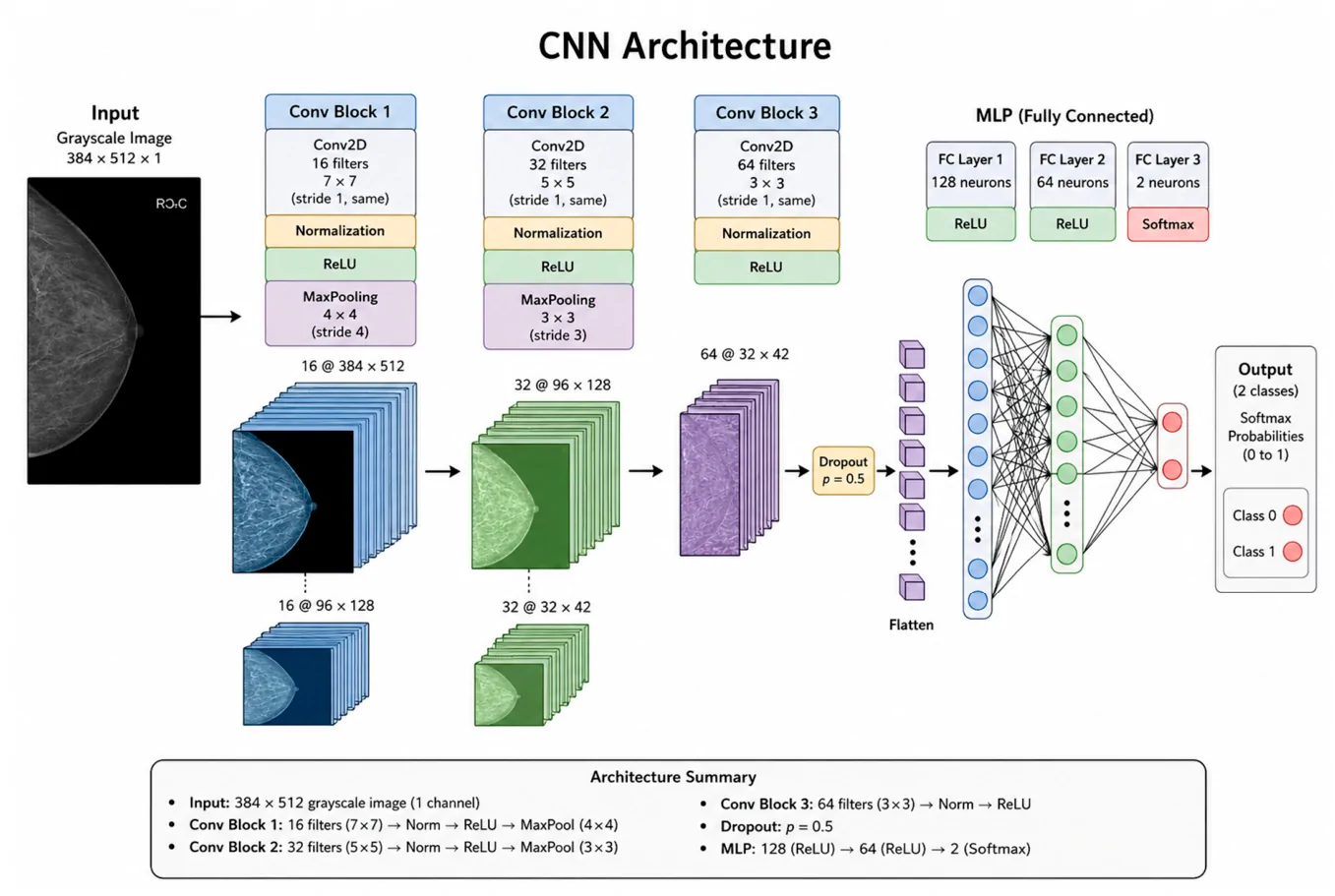
Novel CNN architecture used in this work.

A novel architecture was designed and trained from scratch in this work. Figure 3 shows the architecture. This option was selected after testing several alternatives. All datasets described in Section 2.1 were used to train different instances of this network.

As previously mentioned, the inputs must be grayscale images of size 384 × 512 pixels. The first filter bank consists of 16 filters of size 7 × 7, followed by normalization, ReLU activation, and max pooling with a factor of 4. This is followed by 32 filters of size 5 × 5, normalization, ReLU activation, and max pooling with a factor of 3. The final filter bank contains 64 filters of size 3 × 3, followed by normalization and ReLU activation.

The dropout layer removes output pixels with a probability of 0.5, providing protection against overfitting. The final MLP consists of three fully connected layers with 128, 64, and 2 neurons, respectively.

This architecture is trained using the backpropagation algorithm with the SGDM optimizer, a mini-batch size of 128, and 30 epochs. The optimization target is recall (also called sensitivity, the probability of detecting a positive case). At the beginning of each training process, some samples are set aside for validation during training (1/9 of the training samples), while the test set is not used until the final evaluation.

Training was performed in the same way for the six datasets described in the previous subsection, with the results presented in Section 3. For the AUG-xx datasets, balancing was performed before training by random repetition of minority-class samples.

In all cases, data augmentation was applied to each mini-batch by performing small random transformations on each image to introduce variability into the data. This process helps to avoid overfitting. These transformations do not alter the class of each sample: a small vertical displacement (randomly between −30 and +30 pixels) and a vertical mirror reflection along the Y-axis, applied randomly with a probability of 0.50.

To provide a comparative baseline, an additional CNN architecture was trained using transfer learning with ResNet-18 [18]. This architecture was selected among the currently available pretrained models because of its relative simplicity, with a smaller number of parameters, which is particularly suitable when working with moderately sized datasets. In addition, residual networks have consistently shown strong performance and robustness in medical image classification tasks.

As is standard in transfer learning, the original output layer of ResNet-18 was replaced with a new fully connected layer containing two output neurons corresponding to the two target classes. This final layer was trained using a higher learning rate than the pretrained layers in order to adapt the network to the specific classification problem.

Since ResNet-18 requires RGB input images of size 224 × 224 pixels, the preprocessed mammograms were resized accordingly and converted from grayscale to RGB by replicating the single intensity channel across the three color channels. The same training strategy described previously was applied in this case to ensure a fair comparison with the proposed custom CNN architecture.

### 2.5. Second Classification Stage

This second stage uses the outputs (logits) of the CNNs described in the previous subsection as a feature vector to make the final decision. The idea is to apply N networks to the same image and obtain six numerical values. Note than N can range from 2 to 12.

The main reason for implementing this second stage was the limited performance achieved by the individual CNN models, which by themselves were not sufficient to provide the level of diagnostic accuracy required for a reliable clinical support system. Although the CNNs were able to extract relevant predictive information from the mammographic images, their individual classification performance remained moderate. Therefore, an additional classification stage was necessary to improve the overall system performance by combining the outputs of CNN networks into a single feature vector. This ensemble strategy allows the model to take advantage of complementary information provided by each CNN, resulting in better generalization, higher robustness, and improved sensitivity for breast cancer detection.

Since a softmax activation function is used at the end of each CNN (Figure 3), the two output neurons always produce numerical outputs in probability format (between 0.0 and 1.0, with a total sum equal to 1.0). By taking the output corresponding to the “Sick” class, we obtain something similar to a probability of disease detected by that network. The other logit (“Healthy” class) could be used, but as two logit sum is 1.0, it would convey the same information.

It should be noted that in this scheme, an image of type CC (for example) is also processed by networks trained with MLO images. Note that numerical output in this case can be used as a feature vector component. The underlying idea is that the low-level characteristics that define the diagnosis do not depend majorly on the projection type.

Note that previously trained CNNs are now treated as feature extractors, mainly as specialized filters so that a new classification problem is raised.

The methods evaluated were:

- A simple multilayer perceptron (MLP) with one hidden layer and a single output neuron (probability of disease). This neural network was optimized in two ways. First, the hidden layer size was determined by performing a preliminary training with an excessive number of neurons and analyzing the autocorrelation matrix of the hidden-layer outputs and its eigenvalues. Then, once trained, the best output threshold for declaring a positive case was selected by maximizing the parameter: $F_{\beta }=1/[\frac{1}{1+\beta^{2}}\left(\frac{1}{precision}+\frac{\beta^{2}}{recall}\right)]$, where precision is understood as the probability that a positive prediction is correct, and recall as the probability that a true positive case is detected. The parameter *β* determines how strongly recall is prioritized. In this work, *β* = 1.5 was used.
- A support vector machine (SVM) model [19], which consists of defining a transformation of the feature vectors that simplifies the classification problem by making it linearly separable. In this case, an automatic parameter optimization option was applied.
- A bagged tree model (bootstrap aggregated trees, BT) [20] with ten trees. In this case, several decision trees are trained using random (and overlapping) subsets of the training set. The final decision is obtained by running all constructed trees in parallel and averaging their results. For proper training, the cost of a false negative was set to twice the cost of a false positive.
- A gradient boosting model (XGBoost) [21]. This technique is based on the sequential construction of weak models (decision trees), where each new model is trained to correct the errors made by the previous ones (implemented by optimizing a loss function using gradient descent). Automatic parameter optimization was applied, and the cost of a false negative was also set to twice the cost of a false positive.
- The final method tested was an heuristic calculation (HRS). In this case, six disease probability estimates are available. These are converted into health probabilities: *x′* = 1 − *x*. They are then averaged using a harmonic mean instead of an arithmetic mean: $\bar{x}=1/[\frac{1}{n}\sum_{i=1}^{n}\frac{1}{x_{i}}]$ (the inverse of the averaged inverses). The use of this formula causes the average to decrease much more sharply if one of the probabilities decreases compared to a linear average. This average is considered the global probability of health, and therefore: $1-\bar{x}$ is used as the global discriminant function. As in the MLP case, the optimal threshold is again calculated by maximizing *F_β_* with *β* = 1.5.

Note that the idea of this two-stage strategy is allowing the system to combine the feature extraction capability of convolutional neural networks with the robustness of classical machine learning classifiers. By integrating the outputs of multiple CNN models trained under different conditions, the final classifier benefits from complementary predictive information, leading to improved generalization and higher diagnostic reliability. This ensemble approach is particularly suitable for medical decision-support systems, where maximizing sensitivity and reducing false negatives are critical requirements.

### 2.6. Application of Feature Engineering Techniques

Two feature engineering techniques were applied to assist in selecting the most appropriate CNNs from the 12 available models. Feature engineering (or feature analysis) involves assessing the relevance of each component of the feature vector (i.e., each feature or table column) while also identifying potential dependencies among features. This analysis can simplify the feature vector by eliminating redundant or non-informative features, thereby reducing complexity and potentially improving the performance of the final classifier.

Among the available feature selection approaches, filter-based methods are particularly suitable for this application because they evaluate the importance of each feature independently of the classification model. This allows feature relevance to be determined before model training. Two of the most widely used filter methods are ANOVA and the Pearson correlation coefficient.

In ANOVA (analysis of variance), the idea is that a feature is good when samples are split according to desired outputs and we get sets with different mean and small variance. A measure of importance is computed for each feature with mean differences in the numerator and a variance-like formula in the denominator [22].

The Pearson correlation coefficient is a statistical measure ranging from −1 to 1 that quantifies the strength and direction of the linear relationship between two variables. It is computed from the covariance and variances of the variables [23]. The larger the absolute value of the coefficient, the stronger the linear association. Consequently, the correlation between each feature and the target output can be used as an indicator of feature relevance.

## 3. Results

### 3.1. Performance Metrics

The results obtained for each of the trained models are presented below. For each case, performance metrics were calculated using the test subset of the corresponding dataset. These evaluation metrics are defined as follows:

(1) $$P=precision=\frac{TrueDetections}{TrueDetections+FalseDetections}\;R=recall=\frac{TrueDetections}{TrueDetections+MissedDetections}\;F_{1}=\frac{2\cdot P\cdot R}{P+R}$$

These metrics were selected because they provide a meaningful evaluation of classification performance in medical diagnosis problems, where the cost of errors is not equally distributed. Precision measures the reliability of positive predictions, indicating how many detected cancer cases are actually correct, which helps reduce unnecessary follow-up procedures and patient anxiety caused by false positives. Recall (or sensitivity) is particularly important in this context because it measures the ability of the system to correctly identify true positive cases, minimizing false negatives, which represent missed cancer diagnoses and may have serious clinical consequences. The F_1_ score combines both precision and recall into a single metric, providing a balanced assessment of overall performance. Since breast cancer screening systems must prioritize the detection of positive cases while maintaining acceptable precision, these three metrics are more informative and clinically relevant than overall accuracy alone, especially when working with imbalanced datasets.

### 3.2. CNN Results (First Stage)

**Figure 4:**
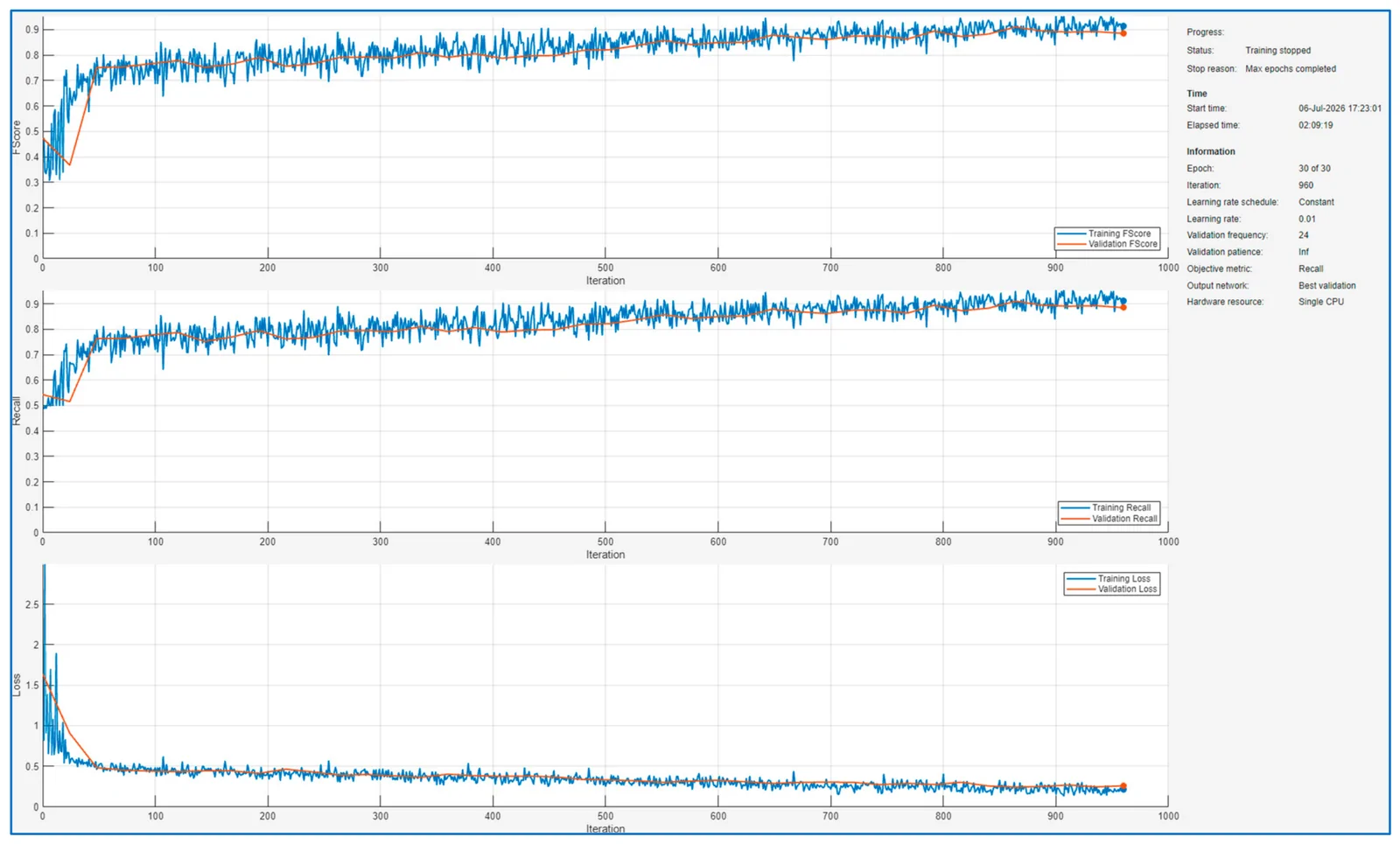
Training curves for AUG-MLO.

The results obtained for the different trained CNNs are presented in Table 3 (using the novel architecture described in Section 2.4). Figure 4 illustrates the training of AUG-CC network (all training curves are similar). Note both training and validation curves arrive together to the final result without overfitting symptoms.

From the table, it can be concluded that none of these models is sufficient when used individually. It should be noted that up to this point, the models have been tested using the same type of images as those in their respective training datasets. Therefore, BLD-CC should only be used with CC mammograms, while AUG-MLO should be used with MLO-type mammograms.

Pretrained networks using the ResNet-18 architecture are customized via transfer learning and assessed, obtaining the results presented below (Table 4).

The results obtained are better than those of the previously proposed architecture, but it is still convenient to apply some kind of improvement. Therefore, it is worthwhile to evaluate both CNN architectures together as feature extractors for the second-stage classifier, with the aim of determining whether combining their outputs can further improve the overall diagnostic performance.

### 3.3. Second-Stage Results

Two types of training were conducted at this stage. First, projection-specific training was performed using a single mammographic projection, either CC or MLO. For the CC projection, the BLD-CC dataset was used, and the first-stage CNNs were selected from the following models: BLD-CC, BLD-MIX, AUG-CC, AUG-MIX, BLD-CC(r18), BLD-MIX(r18), AUG-CC(r18), and AUG-MIX(r18). Each network is named after the dataset on which it was trained, while the suffix “(r18)” denotes models based on the ResNet-18 architecture. For the MLO projection, the BLD-MLO dataset was used, and the CNNs were selected following the same procedure.

The second training strategy combined both CC and MLO projections, allowing the second-stage classifier to select features from all 12 available first-stage CNNs.

For each training configuration (CC-specific, MLO-specific, and mixed projections), two experiments were conducted. In the first, all available first-stage CNNs were included. In the second, a subset of CNNs was selected using the feature engineering techniques described in Section 2.6. It should be noted that both the Pearson correlation coefficient and ANOVA require the outputs of all CNNs to be computed beforehand, as feature selection is performed prior to training the second-stage classifier.

The following tables summarize the results obtained for all six experimental configurations—CC, MLO, and mixed projections—each evaluated with and without feature selection. The presentation of the results begins with the CC projection, whose performance is reported in Table 5.

**Figure 5:**
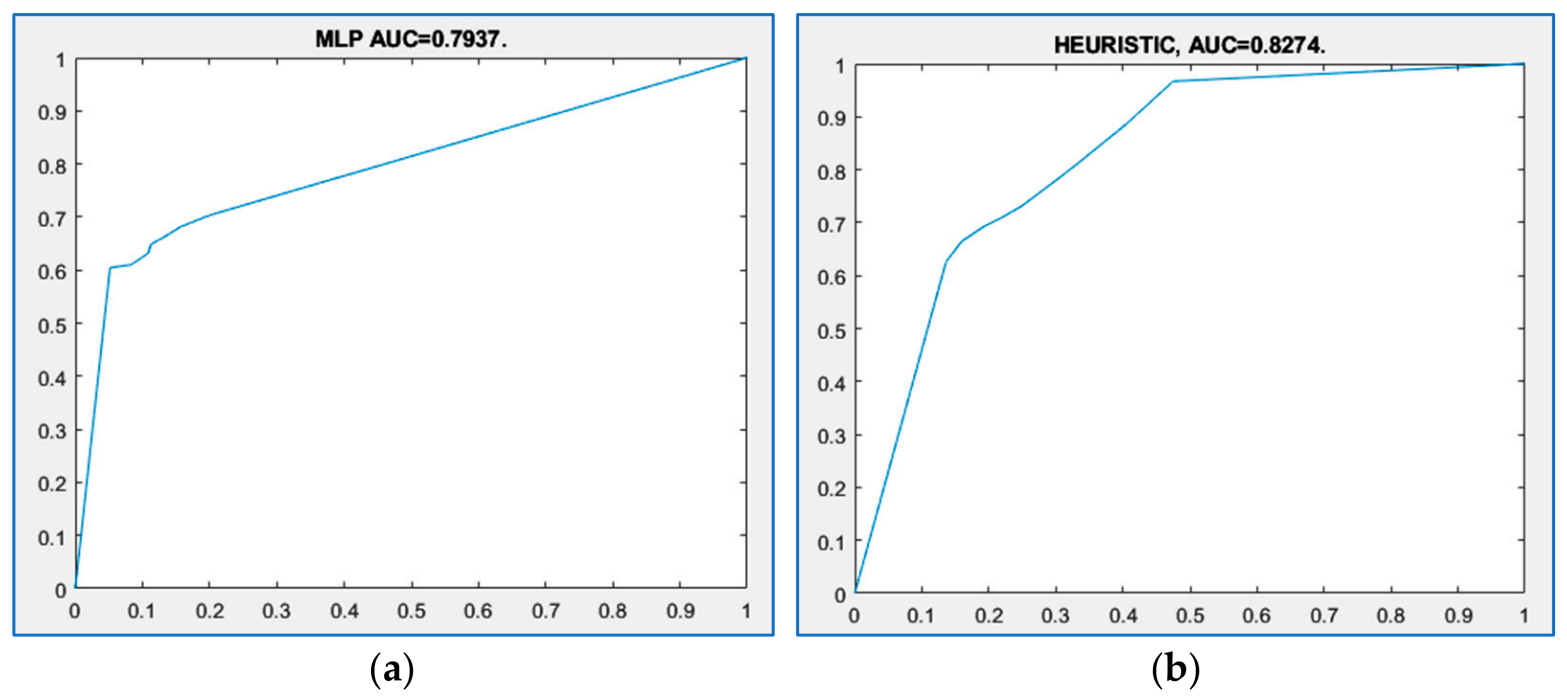
(**a**) ROC curve and AUC value for MLP. (**b**) ROC curve and AUC value for HRS.

Note that AUC (area under the curve) values are computed only for MLP and HRS because the other machine learning schemes provide a binary result. ROC curves are computed for MLP and HRS in all training. Figure 5 shows the curves corresponding to this example. The bigger the area under the curve, the better the system performance.

**Figure 6:**
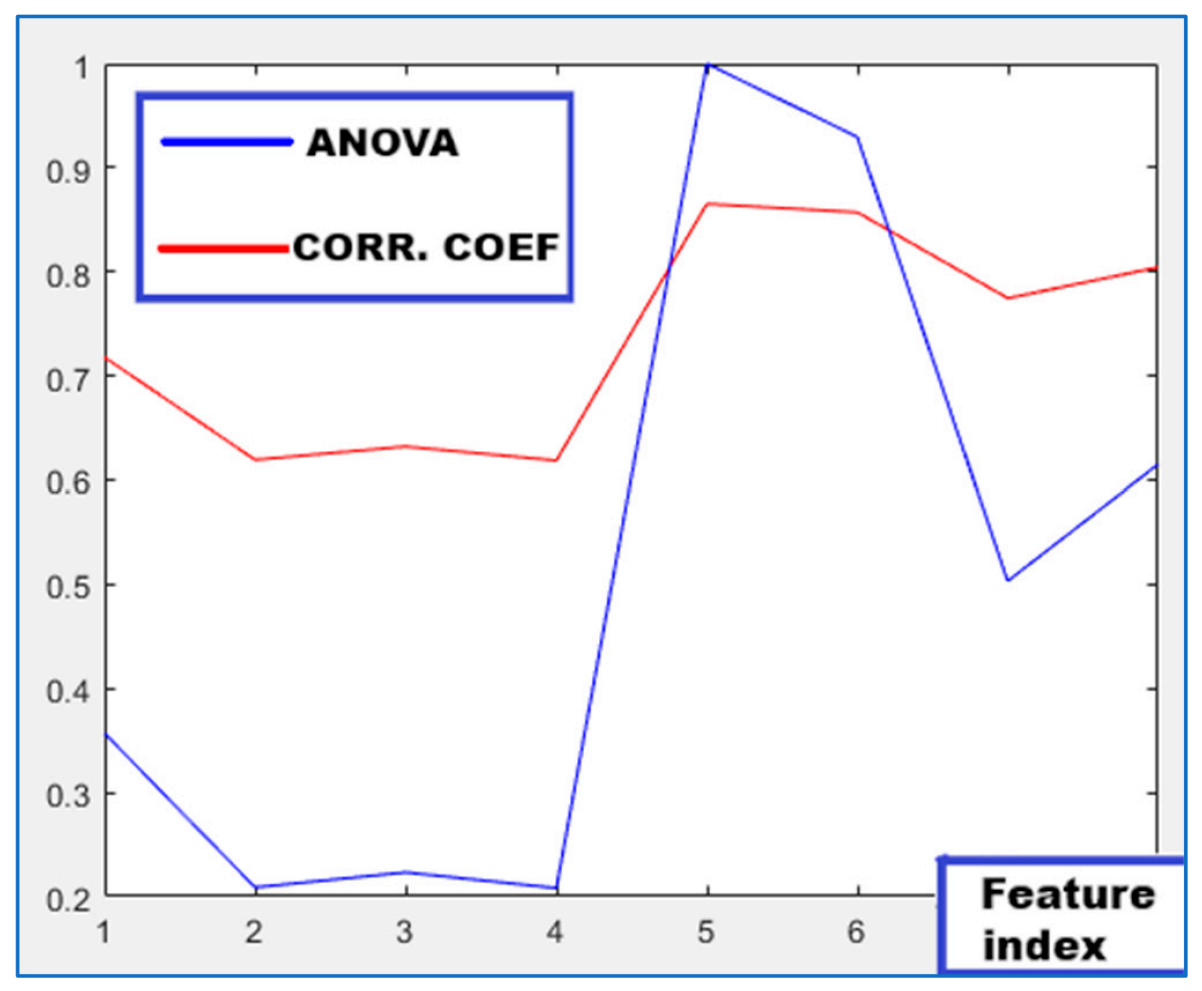
ANOVA and Pearson coefficients for CC projection.

Results of feature analysis for the CC training are shown in Figure 6 below. Bigger values of both curves mean more discriminant features for the second stage. Note as both criteria yield basically the same result. Analyzing the curves, features from the CNNs—BLD-CC(r18), BLD-MIX(r18), AUG-CC(r18), and AUG-MIX(r18)—are selected.

With the reduction new results are obtained and summarized in Table 6 below.

Results for projection MLO without selection are provided below in Table 7.

**Figure 7:**
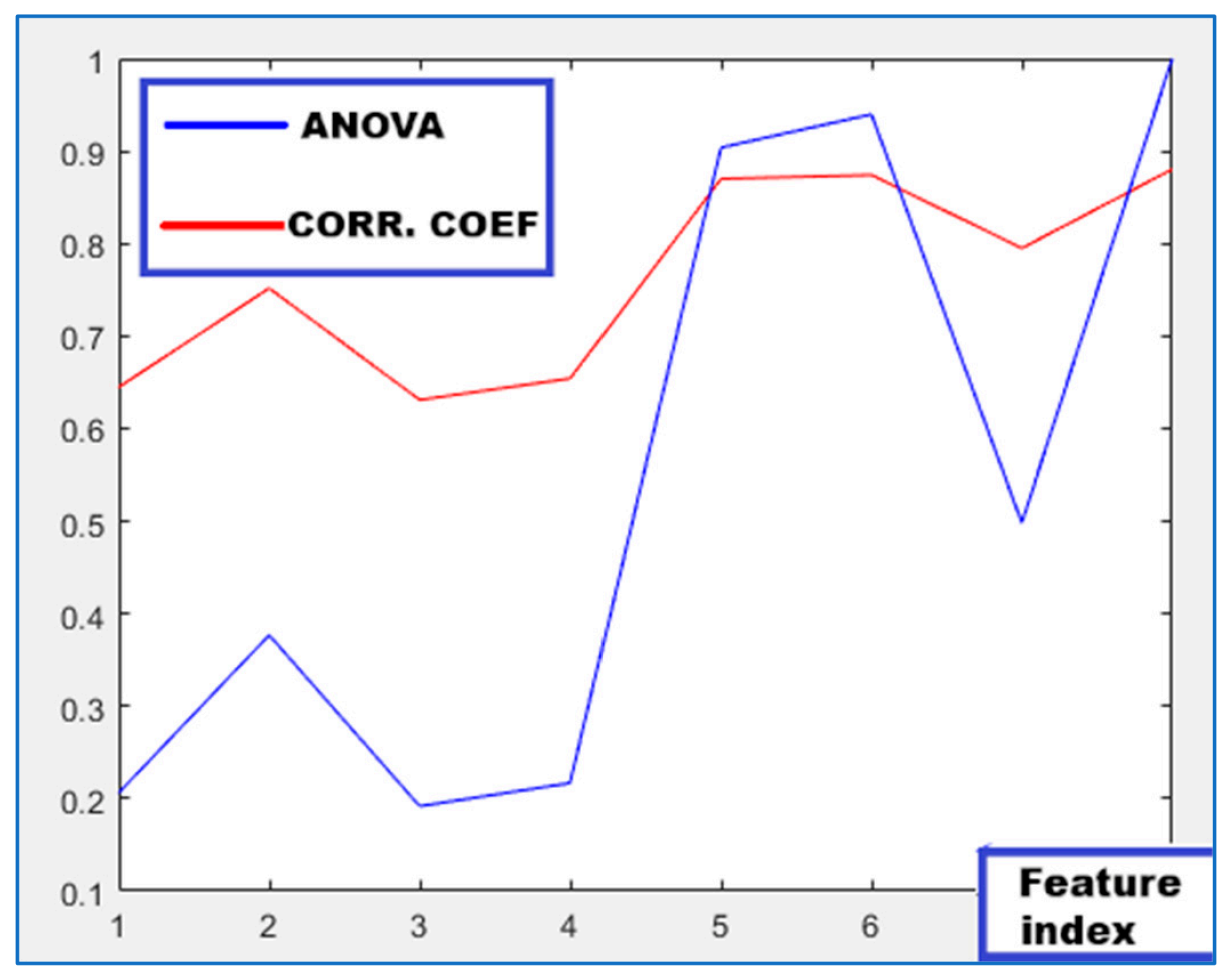
ANOVA and Pearson coefficients for MLO projection.

Results of feature analysis for the MLO training are shown in Figure 7. Analyzing the curves, features from the CNNs—BLD-MLO(r18), BLD-MIX(r18), AUG-MLO(r18), and AUG-MIX(r18)—are selected.

With the reduction new results are obtained and summarized in Table 8 below.

Results mixing projections without feature selection are provided below in Table 9.

**Figure 8:**
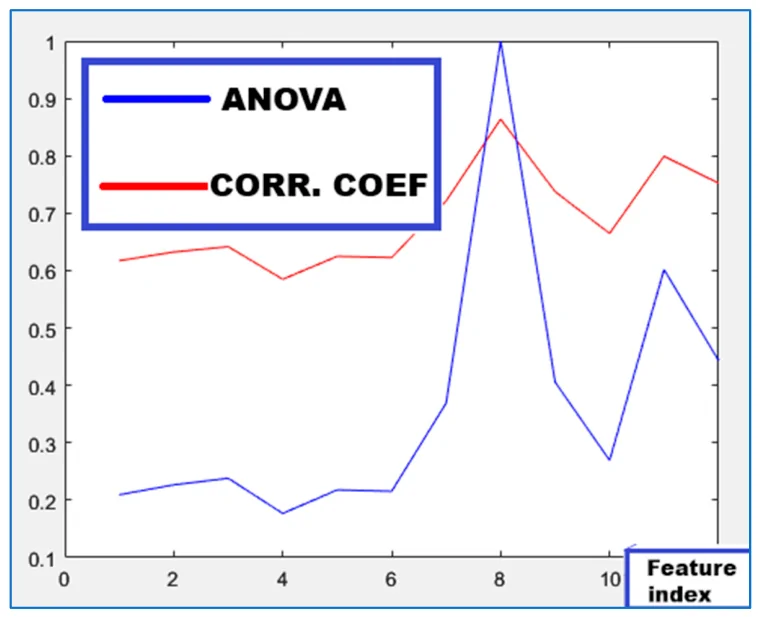
ANOVA and Pearson coefficients for mixed projection.

Results of feature analysis for the MLO training are shown in Figure 8. Analyzing the curves, features from the CNNs—AUG-CC(r18), AUG-MLO(r18), AUG-MIX(r18), BLD-MLO(r18), and BLD-MIX (r18)—are selected.

With the reduction, new results are obtained and summarized in Table 10 below. Note that after applying reduction techniques only CNNs with ResNet18 architecture are retained (with better results in Table 3 and Table 4).

Overall, the experimental results demonstrate that the proposed two-stage ensemble strategy consistently improves upon the performance of the individual CNN models. While the standalone networks provide only moderate classification performance, their combination through a second-stage classifier yields a more robust and reliable diagnostic system. Among the evaluated methods, the MLP classifier offers the best balance between precision and recall, whereas the heuristic fusion approach achieves the highest sensitivity at the expense of precision, making it an interesting alternative for applications where minimizing false negatives is the primary objective. Feature selection based on ANOVA and Pearson correlation further simplifies the ensemble while maintaining, and in some cases slightly improving, the overall performance. These results confirm that combining complementary information extracted by multiple CNNs constitutes an effective strategy for improving breast cancer diagnosis from digital mammograms.

### 3.4. K-Fold Experiment

To evaluate the robustness of the proposed second-stage classifiers, a 10-fold cross-validation experiment was performed on each logits dataset, maintaining the test that was not used to train the first stage. In each iteration, the dataset was partitioned into ten subsets, with one subset left out of the training. The standard deviations of the different figures of merit (FoMs) were then computed across the ten folds, providing an estimate of the stability and robustness of the proposed methodology.

Table 11, Table 12 and Table 13 summarize the standard deviations obtained for the reduced feature sets selected through the ANOVA procedure described in the previous subsection. The low standard deviation values indicate that the proposed approach provides stable performance and exhibits good generalization across different training and testing partitions.

The consistently low standard deviation values (almost all below 0.05, except XGBoost in Table 11) confirm that the proposed methodology exhibits good robustness and generalization, with only limited performance variability across the different cross-validation folds.

### 3.5. Dataset Ablation Experiment

The combination of two datasets, where the second dataset contains only positive cases, raises the possibility that the model may overfit to characteristics specific to that dataset rather than learning disease-related features. Although both datasets are highly heterogeneous, an additional experiment was designed to evaluate this potential source of bias.

The proposed experiment is based on dataset ablation. All samples from the second dataset were removed, and the six working datasets were reconstructed using only the remaining data. Consequently, the balanced training datasets contained approximately 400 samples per class. For the augmented datasets, the negative training samples remained unchanged, while the number of positive training samples was limited to 1000 to avoid excessive oversampling.

As expected, reducing the amount of available training data is likely to degrade the overall classification performance. However, if the relative behavior of the different second-stage classifiers remains consistent with the previous experiments, this would indicate that the proposed approach does not rely on dataset-specific characteristics and therefore generalizes appropriately.

The results are presented in Table 14, which reports the F_1_ score obtained by each second-stage classifier. Each column corresponds to one of the configurations evaluated in the previous subsections.

As anticipated, Table 14 exhibits lower F_1_ scores than those obtained with the complete datasets due to the reduced amount of training data. Nevertheless, the overall performance trends remain consistent with the previous experiments, suggesting that the proposed methodology is robust and that its performance is not primarily driven by characteristics unique to the removed dataset.

### 3.6. Demonstration Application

A demonstration application has been created that allows the estimation of cancer risk from an image that can be provided in JPEG or DICOM format. This involves running the entire process: preprocessing, N CNN networks, feature vector creation, and execution of the models trained in the previous subsection.

A MODELs folder has to be provided where the user must copy the files defining the CNNs to be used and also the second-stage models. App will use all the models found. For MLP and HRS (if available), a risk percentage between 0 and 100 is computed, taking into account not only the output login but also the threshold that was computed in the training phase.

**Figure 9:**
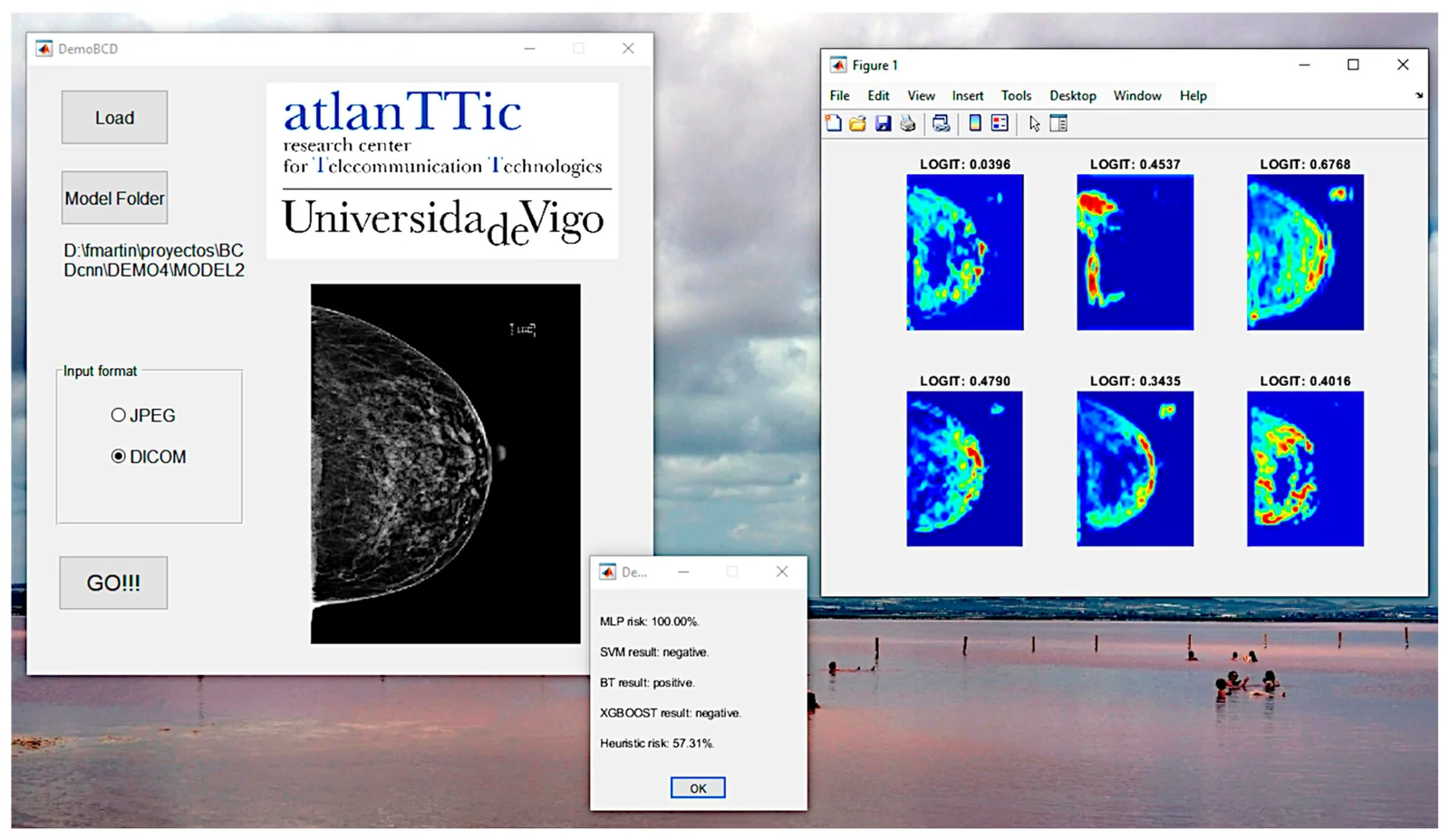
Screenshot of the DEMO app including the Grad-Cam activation maps.

As a final enhancement of the application, an explainability option was incorporated to allow users to better understand the diagnostic results using Grad-CAM [24]. Grad-CAM (gradient-weighted class activation mapping) is a visualization technique used to explain the decisions made by CNNs by analyzing the gradients of the target class (the “Sick” class in this case) with respect to the feature maps of the last convolutional layer. Based on this information, a heatmap is generated to indicate how strongly each region of the input image contributes to the final prediction. This functionality is particularly valuable in positive cases, as it helps verify whether the model is focusing on clinically relevant suspicious areas of the mammogram, improving interpretability and increasing confidence in the diagnostic output. App windows including feature maps for a real positive case are shown in Figure 9. Find on the left the main application, in the center the results dialogue and on the right the heat maps for Grad-Cam.

## 4. Discussion

This work has presented a two-stage ensemble framework for breast cancer diagnosis from digital mammograms, combining the predictions of multiple convolutional neural networks (CNNs) through a second-stage machine learning classifier. The experimental results demonstrate that this strategy consistently outperforms the individual CNN models, confirming that the information extracted by independently trained networks is complementary and can be effectively exploited by a higher-level classifier [9,10,11].

The first-stage CNNs exhibited moderate classification performance when used individually. As expected, the ResNet-18 models [18] trained by transfer learning generally achieved better results than the custom CNN architecture, indicating that the knowledge acquired from large-scale natural image datasets remains beneficial after fine-tuning for mammographic image analysis. However, neither architecture alone provided the best overall performance, suggesting that different networks learn distinct characteristics of the data. The proposed ensemble successfully combines these complementary representations, resulting in improved classification accuracy and robustness.

Among the second-stage classifiers, the multilayer perceptron (MLP) consistently provided the most balanced performance in terms of precision, recall, F_1_ score, and AUC, making it the preferred option for the proposed framework. The support vector machine (SVM) also achieved competitive results, particularly in terms of precision, whereas the bagged trees classifier showed a slightly lower but still stable performance. The heuristic fusion rule based on harmonic means yielded the highest recall values, although at the expense of a reduction in precision due to an increased number of false positives. Such behavior may nevertheless be advantageous in breast cancer screening applications, where minimizing false negatives is often considered more important than reducing false alarms [8].

The feature selection stage [22,23] based on ANOVA proved effective in reducing the dimensionality of the second-stage input while maintaining, and in some cases slightly improving, the classification performance. The selected subsets were dominated by the outputs of the ResNet-18 models, indicating that these classifiers contributed most of the discriminative information available to the ensemble. Furthermore, the 10-fold cross-validation experiments showed consistently low standard deviations for all evaluated figures of merit, demonstrating that the proposed methodology is robust and that its performance is not strongly dependent on a particular training and testing partition.

Despite these encouraging results, several limitations should be acknowledged. First, the proposed framework was evaluated using publicly available datasets, which although widely adopted in the literature, may not fully represent the variability encountered in routine clinical practice. Second, the datasets remain relatively limited in size compared with those available for other computer vision applications, potentially restricting the generalization capability of deep learning models. Third, the study focused on binary classification of normal and abnormal mammograms without considering lesion localization, subtype classification, or integration with additional clinical information. Fourth, the computational cost associated with training multiple CNNs, though incurred only during the training stage, is higher than that of a single-network solution. Finally, it would be extremely interesting getting images from local healthcare institutions for further testing and validation.

Quantitative comparison with other similar systems is provided in Table 15 below.

Data for our system correspond to a system with mixed projections and no dataset reduction (Table 9). Note that conditions for the alternative systems are different as, for example, in [6,7] a predefined ROI is provided and [8] uses several images of different modalities.

Future work will focus on extending the proposed methodology in several directions. The ensemble could be evaluated using larger multi-institutional datasets to assess its generalization across different imaging devices and acquisition protocols. More recent deep learning architectures, transformer-based models, or self-supervised learning approaches could also be incorporated into the first-stage ensemble. Alternative feature selection techniques and meta-learning strategies may further improve the second-stage classifier, while probability calibration methods could enhance the reliability of the predicted malignancy scores. Finally, integrating explainability techniques such as Grad-CAM with quantitative clinical indicators and evaluating the proposed system in collaboration with radiologists would provide a more comprehensive assessment of its usefulness as a computer-aided diagnosis tool.

In conclusion, the proposed two-stage ensemble constitutes an effective and robust approach for mammographic breast cancer classification. By combining the outputs of multiple CNN models through a second-stage classifier, the framework consistently improves diagnostic performance over individual networks while maintaining stable behavior across different validation folds. The results suggest that heterogeneous ensemble learning represents a promising strategy for enhancing computer-aided diagnosis systems and merits further investigation using larger and more diverse clinical datasets.

## Figures and Tables

**Table 1:** Main dataset.

	CC	MLO	ML	LM
Healthy	26,199	27,313	8	10
Sick	566	590	0	0

**Table 2:** Work datasets.

		BLD-CC	BLD-MLO	BLD-MIX	AUG-CC	AUG-MLO	AUG-MIX
Training	Healthy	1017	1090	2107	2320	2307	4627
	Sick	1015	1090	2105	1015	1090	2105
Testing	Healthy	180	193	373	180	193	373
	Sick	182	193	375	182	193	375

**Table 3:** CNN results (novel architecture of Figure 3).

Training Dataset	Precision	Recall	F_1_
BLD-CC	0.70	0.64	0.67
BLD-MLO	0.98	0.54	0.70
BLD-MIX	0.87	0.59	0.71
AUG-CC	0.77	0.64	0.70
AUG-MLO	0.74	0.67	0.71
AUG-MIX	0.95	0.53	0.68

**Table 4:** CNN results (ResNet-18).

Training Dataset	Precision	Recall	F_1_
BLD-CC	0.81	0.68	0.74
BLD-MLO	0.83	0.67	0.74
BLD-MIX	0.82	0.68	0.74
AUG-CC	0.85	0.67	0.75
AUG-MLO	0.83	0.64	0.73
AUG-MIX	0.83	0.58	0.68

**Table 5:** Second stage (final) results: CC projection without feature selection.

	MLP	SVM	BT	XGBoost	HRS
Precision	0.89	0.89	0.85	0.85	0.60
Recall	0.63	0.63	0.64	0.63	0.88
F_1_	0.74	0.73	0.73	0.73	0.71
AUC	0.79	-	-	-	0.83

**Table 6:** Second-stage (final) results: CC projection with feature selection.

	MLP	SVM	BT	XGBoost	HRS
Precision	0.90	0.91	0.87	0.88	0.68
Recall	0.67	0.64	0.63	0.66	0.84
F_1_	0.77	0.75	0.73	0.75	0.75
AUC	0.81	-	-	-	0.81

**Table 7:** Second-stage (final) results: MLO projection without feature selection.

	MLP	SVM	BT	XGBoost	HRS
Precision	0.81	0.82	0.80	0.80	0.53
Recall	0.68	0.68	0.69	0.69	0.95
F_1_	0.74	0.74	0.74	0.74	0.68
AUC	0.76	-	-	-	0.80

**Table 8:** Second-stage (final) results: MLO projection with feature selection.

	MLP	SVM	BT	XGBoost	HRS
Precision	0.81	0.83	0.78	0.81	0.66
Recall	0.69	0.68	0.69	0.70	0.83
F_1_	0.74	0.75	0.73	0.75	0.74
AUC	0.76	-	-	-	0.78

**Table 9:** Second-stage (final) results: mixed projections without feature selection.

	MLP	SVM	BT	XGBoost	HRS
Precision	0.83	0.87	0.85	0.85	0.59
Recall	0.69	0.65	0.68	0.66	0.90
F_1_	0.75	0.75	0.76	0.76	0.71
AUC	0.78	-	-	-	0.82

**Table 10:** Second-stage (final) results: mixed projections with feature selection.

	MLP	SVM	BT	XGBoost	HRS
Precision	0.80	0.87	0.84	0.84	0.66
Recall	0.71	0.64	0.68	0.68	0.84
F_1_	0.75	0.74	0.75	0.75	0.74
AUC	0.79	-	-	-	0.78

**Table 11:** Deviation of FoMs for CC projection with feature selection.

	MLP	SVM	BT	XGBoost	HRS
Precision	0.0235	0.0051	0.0114	0.1134	0.0000
Recall	0.0153	0.0116	0.0145	0.1066	0.0000
F_1_	0.0039	0.0077	0.0103	0.0243	0.0000
AUC	0.0052	-	-	-	0.0000

**Table 12:** Deviation of FoMs for MLO projection with feature selection.

	MLP	SVM	BT	XGBoost	HRS
Precision	0.0081	0.0046	0.0133	0.0124	0.0000
Recall	0.0073	0.0000	0.0087	0.0093	0.0000
F_1_	0.0031	0.0019	0.0066	0.0085	0.0000
AUC	0.0076	-	-	-	0.0000

**Table 13:** Deviation of FoMs for mixed projections with feature selection.

	MLP	SVM	BT	XGBoost	HRS
Precision	0.0109	0.0016	0.0080	0.0126	0.0000
Recall	0.0112	0.0061	0.0087	0.0109	0.0000
F_1_	0.0036	0.0043	0.0065	0.0066	0.0000
AUC	0.0049	-	-	-	0.0000

**Table 14:** F_1_ scores obtained using the reduced datasets.

	CC All Features	CC Selected Features	MLO All Features	MLO Selected Features	MIX All Features	MIX Selected Features
MLP	0.56	0.53	0.59	0.58	0.60	0.57
SVM	0.56	0.55	0.55	0.54	0.54	0.54
BT	0.56	0.56	0.58	0.57	0.55	0.53
XGBoost	0.55	0.57	0.58	0.61	0.53	0.55
HRS	0.67	0.67	0.66	0.65	0.67	0.66

**Table 15:** Comparison of results with previous bibliography papers.

	Ours, MLP	Ours, HRS	[5]	[6]	[7]	[8]
Precision	0.83	0.59				0.97
Recall	0.69	0.90				0.98
F_1_	0.75	0.71				0.97
AUC	0.78	0.82	0.88			
IoU	NA	NA		0.87		
Accuracy	0.77	0.63	0.89		0.85	0.97

## Data Availability

The original contributions presented in this study are included in the article. Further inquiries can be directed to the corresponding author.

## Abbreviations

The following abbreviations are used in this manuscript.

AUC	Area Under the Curve
AUG	Augmented dataset
BLD	Balanced dataset
BT	Bagged tree classifier
CAD	Computer aided diagnosis
CC	Cranial caudal view
CNN	Convolutional neural network
DICOM	Digital imaging and communications in medicine
F_1_ score	Harmonic mean of precision and recall
FoM	Figure of Merit
GAN	Generative Adversarial Networks
HRS	Heuristic aggregation method
LM	Latero-medial view
ML	Medio-lateral view
MLO	Medio-lateral oblique view
MLP	Multi-layer perceptron
ReLU	Rectified linear unit
ROC	Receiver Operating Characteristic
ROI	Region of Interest
SGDM	Stochastic gradient descent with momentum
SMOTE	Synthetic minority oversampling technique
SVM	Support vector machine
WHO	World health organization
XGBoost	eXtreme gradient boosting

## References

[1] World Health Organization (WHO) (2021). Breast Cancer.

[2] Lecun Y., Bottou L., Bengio Y., Haffner P. (1998). Gradient-based learning applied to document recognition. Proc. IEEE.

[3] Rumelhart D., Hinton G., Williams R. (1986). Learning Representations by Back-propagating Errors. Nature.

[4] Nasser M., Yusof U.K. (2023). Deep Learning Based Methods for Breast Cancer Diagnosis: A Systematic Review and Future Direction. Diagnostics.

[5] Shams S., Platania R., Zhang J., Kim J., Lee K., Park S.J., Frangi A., Schnabel J., Davatzikos C., Alberola-López C., Fichtinger G. (2018). Deep Generative Breast Cancer Screening and Diagnosis. Medical Image Computing and Computer Assisted Intervention (MICCAI 2018).

[6] Singh V.K., Rashwan H.A., Romani S., Akram F., Pandey N., Sarker M.M.K., Saleh A., Arenas M., Arquez M., Puig D. (2020). Breast tumor segmentation and shape classification in mammograms using generative adversarial and convolutional neural network. Expert Syst. Appl..

[7] Guan S., Loew M. Breast cancer detection using synthetic mammograms from generative adversarial networks in convolutional neural networks. Proceedings of the 14th International Workshop on Breast Imaging (IWBI 2018).

[8] Zheng J., Lin D., Gao Z., Wang S., He M., Fan J. (2020). Deep Learning Assisted Efficient AdaBoost Algorithm for Breast Cancer Detection and Early Diagnosis. IEEE Access.

[9] Dehghan Rouzi M., Moshiri B., Khoshnevisan M., Akhaee M.A., Jaryani F., Salehi Nasab S., Lee M. (2023). Breast Cancer Detection with an Ensemble of Deep Learning Networks Using a Consensus-Adaptive Weighting Method. J. Imaging.

[10] Shah E.A. (2024). Optimizing Breast Cancer Detection with an Ensemble Deep Learning Approach. Int. J. Intell. Syst..

[11] Masud E.A. (2025). From Machine Learning to Ensemble Approaches: A Systematic Review of Mammogram Classification Methods. Diagnostics.

[12] Ali M.D., Saleem A., Elahi H., Khan M.A., Khan M.I., Yaqoob M.M., Farooq Khattak U., Al-Rasheed A. (2023). Breast Cancer Classification through Meta-Learning Ensemble Technique Using Convolution Neural Networks. Diagnostics.

[13] Abdikenov B., Zhaksylyk T., Imasheva A., Orazayev Y., Karibekov T. (2025). Innovative Multi-View Strategies for AI-Assisted Breast Cancer Detection in Mammography. J. Imaging.

[14] Jeba Prasanna Idas S., Hemalatha K., Naveenkumar J., Joshva Devadas T. (2025). Recent trends on mammogram breast density analysis using deep learning models: Neoteric review. Artif. Intell. Rev..

[15] Carr C., Kitamura F., Partridge G. RSNA Screening Mammography Breast Cancer Detection. https://www.kaggle.com/code/sarashahin/rsna-screening-mammography-breast-cancerdetection.

[16] National Electrical Manufacturers Association (2026). Digital Imaging and Communications in Medicine (DICOM) Standard.

[17] https://www.kaggle.com/competitions/rsna-breast-cancer-detection/discussion?sort=undefined.

[18] He K., Zhang X., Ren S., Sun J. Deep Residual Learning for Image Recognition. Proceedings of the 2016 IEEE Conference on Computer Vision and Pattern Recognition (CVPR).

[19] Cortes C., Vapnik V. (1995). Support-vector networks. Mach. Learn..

[20] Breiman L. (1998). Bagging predictors. Mach. Learn..

[21] Friedman J.H. (2001). Greedy function approximation: A gradient boosting machine. Ann. Stat..

[22] Tadist K., Najah S., Nikolov N.S., Mrabti F., Zahi A. (2019). Feature selection methods and genomic big data: A systematic review. J. Big Data.

[23] Rahman A., Zaman S., Parvej S., Abdul Fattah H.M. Heart Disease Prediction Using Ensemble Solutions and Exploring the Effect of Target Engineering with Pearson Correlation Based Feature Selection. Proceedings of the 6th International Conference on Electrical Engineering and Information & Communication Technology (ICEEICT).

[24] Selvaraju R.R., Cogswell M., Das A., Vedantam R., Parikh D., Batra D. Grad-CAM: Visual Explanations from Deep Networks via Gradient-Based Localization. Proceedings of the 2017 IEEE International Conference on Computer Vision (ICCV).

